# EXPLORING PREDICTORS OF LONGEVITY USING RETROSPECTIVELY CODED AUTOBIOGRAPHICAL DATA

**DOI:** 10.1093/geroni/igad104.3020

**Published:** 2023-12-21

**Authors:** Brianna Barnett, Alexander Bishop

**Affiliations:** Oklahoma State University, Stillwater, Oklahoma, United States; Oklahoma State University, Stillwater, Oklahoma, United States

## Abstract

The aim of this study was to identify predictors of longevity using retrospectively coded autobiographical stories written and recorded from N = 1,858 deceased centenarians (M = 102.79 years; SD = 2.25 years) from the state of Oklahoma. Using the Developmental Adaptation Model as a conceptual framework, total number of years lived, the developmental outcome was regressed on socio-demographic characteristics including sex, race, and education, as well as retrospectively coded variables reflecting parental occupation, total years married, age at retirement, engagement in international travel, and self-attributions of longevity. Results confirmed three key predictors of living to 100 years and beyond. First, race was confirmed as a strong predictor of longevity (β = −.65, p < .001). Fatherhood agricultural occupation emerged as second key predictor of living 100 years and longer (β = .42, p < .10). Finally, total years spent in a marriage represented a third predictor of longevity (β = .47, p < .01). Results suggest being a person of color, being raised by a father who made a livelihood working in the agricultural industry, and remaining within a long-term marital union are contributing variables linked to living 100 years and longer. Further detail regarding descriptive and methodological evaluation of retrospectively coded centenarian biographies will be highlighted. Results have implications relative to how gerontological researchers and aging service professionals may evaluate and link autobiographical information of long-lived adults to developmental outcomes such as longevity.

